# Acidic pH of early endosomes governs SARS-CoV-2 transport in host cells

**DOI:** 10.1016/j.jbc.2024.108144

**Published:** 2024-12-26

**Authors:** Perla Fares, Mariam Duhaini, Suvranta K. Tripathy, Ali Srour, Kalyan C. Kondapalli

**Affiliations:** Department of Natural Sciences, University of Michigan-Dearborn, Dearborn, Michigan, USA

**Keywords:** sodium-proton exchange, SARS-CoV-2, COVID-19, NHE9, SLC9A9, pH, endocytosis, endocytic trafficking

## Abstract

Endocytosis is a prominent mechanism for SARS-CoV-2 entry into host cells. Upon internalization into early endosomes (EEs), the virus is transported to late endosomes (LEs), where acidic conditions facilitate spike protein processing and viral genome release. Dynein and kinesin motors drive EE transport along microtubules; dynein moves EEs to the perinuclear region, while kinesins direct them towards the plasma membrane, creating a tug-of-war over the direction of transport. Here, we identify that the luminal pH of EEs is a key factor regulating the outcome of this tug-of-war. Among the known endosomal pH regulators, only the sodium-proton exchanger NHE9 has so far been genetically linked to severe COVID-19 risk. NHE9 functions as a proton leak pathway specifically on endosomes. We show that limiting acidification of EEs by increasing the expression of NHE9 leads to decreased infectivity of the SARS-CoV-2 spike-bearing virus in host cells. Our investigation identified the EE membrane lipid phosphatidylinositol-3-phosphate (PI3P) as a link between luminal pH changes and EE transport. Normally, as EEs mature, PI3P depletes. However, in cells with high NHE9 expression, PI3P persists longer on EEs. PI3P plays a pivotal role in the recruitment of motor proteins and the subsequent movement of EEs. Consistently, we observed that NHE9-mediated alkalization of EEs hindered perinuclear movement. Specifically, EE speed and run length were negatively impacted, ultimately leading to EEs falling off microtubules and impairing the delivery of viral cargo to LEs. NHE9 thus offers a unique opportunity as a viable therapeutic target to impede SARS-CoV-2 host cell entry.

Severe acute respiratory syndrome coronavirus 2 (SARS-CoV-2) continues to pose a public health threat due to its rapid evolution (1, 2, 3). This underscores the importance of a comprehensive understanding of its infection mechanisms. Efficient infection is characterized by SARS-CoV-2’s ability to enter host cells and utilize the cellular machinery to replicate its genome and produce viral proteins (4). Preventing virus entry is thus a strategic point of intervention.

Receptor-mediated endocytosis is a crucial mechanism for SARS-CoV-2 to enter host cells (5, 6, 7, 8). Entry into host cells is initiated when the virus attaches to the ACE2 receptor, triggering endocytosis (6, 8, 9). The virus is then encapsulated and transported through early endosomes, which progressively acidify as they shuttle the virus past cellular obstacles like cytoplasmic crowding and cortical microfilament networks and deliver it to the late endosomes (8, 10).

pH plays a crucial role in the endocytic route for SARS-CoV-2 entry into host cells. Currently, two steps within the endocytic pathway are recognized as dependent on the pH of the endosomal lumen: the activation of proteases that prime the spike protein, and the facilitation of fusion between the viral and endosomal membranes, which leads to the release of the viral genome (8). However, these stages may not fully capture the complexity of viral trafficking within host cells. Recent observations suggest that impairing luminal acidification disrupts endo-phagosome movement in macrophages, implying an additional role for luminal pH in trafficking between early and late endosomes (11). This observation prompted us to investigate whether endosomal acidification influences similar processes in SARS-CoV-2 trafficking in host cells. Moreover, the existing knowledge on the role of pH in viral entry has largely relied on research using non-selective agents such as ammonium chloride and chloroquine, or treatments with low pH media (5, 8, 12). These methods lack the specificity necessary to distinguish between the different stages of viral entry. This oversight marks a substantial gap in the methodology. Additionally, the lack of actionable targets within the endocytic pathway has stifled advancements in therapeutics.

Endosomal acidification is achieved by the active pumping of protons (H^+^) into the lumen of the endosome *via* the V-ATPase (13). This acidification is finely modulated by a balance of counter-ion fluxes and H^+^ leakage pathways that operate alongside the V-ATPase (14). Among the proteins involved, NHE9, a sodium-proton exchanger specific to endosomes, stands out as a key regulator (15, 16, 17, 18, 19). Notably, the SLC9A9 gene, which encodes NHE9, is the only endosomal pH regulatory protein that has been genetically linked to an increased risk of COVID-19 (20). Function variants of NHE9 are associated with increased susceptibility to SARS-CoV-2 infections (20). However, the mechanisms connecting NHE9-regulated pH changes within the endosome and SARS-CoV-2 infectivity are not known. In this study, we directly manipulated NHE9 expression through genetic perturbations, specifically altering endosomal pH. This targeted change in luminal pH allowed us to discover a critical link between NHE9 expression and the transport of viral cargo from early to late endosomes. Our data suggest that this mechanism operates through changes in phosphatidylinositol-3-phosphate (PI3P) levels in early endosomes. PI3P is involved in the recruitment and regulation of motor proteins on early endosomes, which are essential for cargo transport. Given that endosomal pH is a critical factor in the entry of many viruses, targeting pH regulation could represent a broader therapeutic strategy, extending beyond SARS-CoV-2 to other pathogens that exploit this pathway for entry. Our findings reveal a new upstream role for endosomal pH in the viral entry process, beyond the known steps of protease activation and membrane fusion. This provides new insights into the mechanisms of SARS-CoV-2 entry and identifies NHE9 as a potential target for therapeutic intervention.

## Results

### NHE9 impacts viral cargo delivery from early endosomes to late endosomes

**Figure 1:**
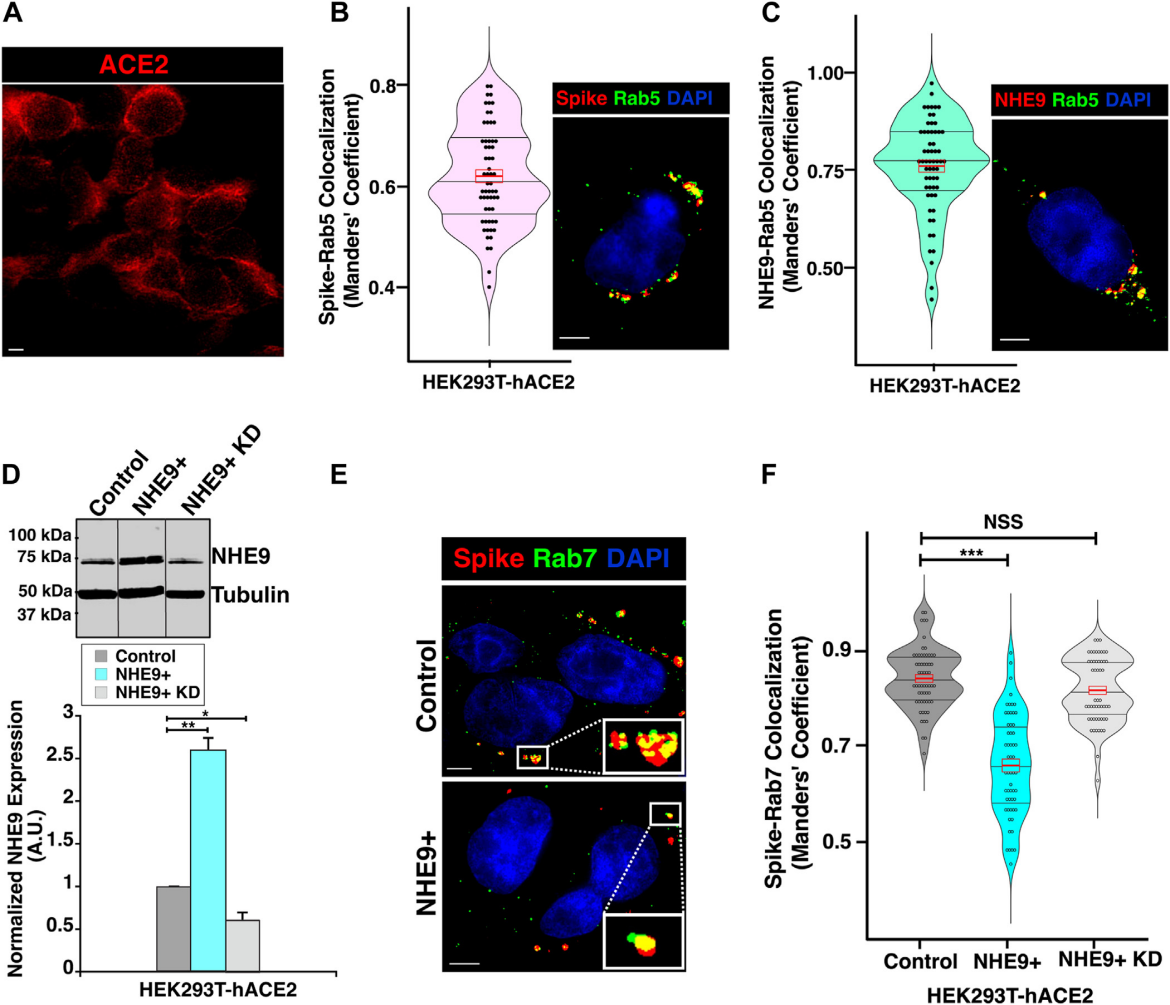
**NHE9 expression impedes the transport of viral cargo to late endosomes.***A*, representative immunofluorescence microscopy image depicting ACE2 expression in HEK293T cells engineered for constitutive ACE2 receptor expression (HEK 293T-hACE2). Scale bar (*white line*) is 10 μm. *B*, *left panel*: Violin plot showing the distribution of Manders’ overlap coefficient values, with a red rectangle indicating the mean (*center line*) and standard deviation (*top and bottom of the rectangle*). This quantifies the colocalization of the spike protein with Rab5 after 5 minutes of internalization. The data represents the mean of two biological replicates, each with at least 30 cells. *Right panel*: Representative immunofluorescence microscopy image showing the colocalization of the spike protein (*red*) with the early endosomal marker Rab5 (*green*) 5 minutes after spike protein internalization. Colocalization is indicated by *yellow dots*. DAPI staining of the nucleus is shown in *blue*. The scale bar (*white line*) represents 5 μm. *C*, same as panel B, except depicting the colocalization of NHE9 with Rab5. *D*, *Top panel*: Immunoblot showing NHE9 expression in HEK293T-hACE2 cells for Control, NHE9 overexpressed (NHE9+), and NHE9 knocked-down in the overexpressing cells (NHE9+ KD). *Bottom panel*: Graphical representation of the average band intensity from densitometric scans of immunoblots for three biological replicates. Error bars indicate standard deviation (SD). Statistical significance is denoted by ∗*p* < 0.05 and ∗∗*p* < 0.005, analyzed using Student’s *t* test. *E*, Representative immunofluorescence microscopy images comparing the colocalization of the spike protein (*red*) with the late endosomal marker Rab7 (*green*) between control (*top panel*) and NHE9+ (*bottom panel*) HEK293T-hACE2 cells, captured 20 minutes post spike protein internalization. Colocalization is indicated by *yellow dots*. DAPI staining of the nucleus is shown in blue. The scale bar (*white line*) represents 5 μm. *F*, quantitative comparison of the colocalization of spike protein with Rab7 between control, NHE9+, and NHE9+ KD cells, captured 20 minutes after internalization of the spike protein. Violin plots show the distribution of Manders' overlap coefficient values, with red rectangles indicating the mean (*center line*) and standard deviation (*top and bottom of the rectangles*). This quantifies the colocalization of the spike protein with Rab7. The data represents the mean of two biological replicates, each consisting of at least 30 cells. Error bars represent standard deviation (SD). ∗∗∗*p* < 0.001. NSS, Not statistically significant. Statistical analysis was done using Student’s *t* test.

Multiple endocytic pathways facilitate SARS-CoV-2 uptake, all converging at the early endosomes on the cell periphery (9, 12). Subsequently, the virus is transported to late endosomes located near the nucleus (8). To study this process, we conducted colocalization analyses using purified SARS-CoV-2 spike protein. This protein was fluorescently labeled with Alexa Fluor 647 to enable visualization post-endocytosis. As previously reported, receptor binding and initial trafficking are likely independent of whether the spike protein is part of a viral particle (9, 21). We incubated purified spike protein with HEK293T cells expressing ACE2 (HEK293T-hACE2, Fig. 1*A*) at 4 °C for 30 minutes to facilitate binding. Subsequently, warming the cells to 37 °C initiated internalization, allowing us to track the spike protein’s progress to early and late endosomes. Within 5 minutes of internalization, the spike protein significantly colocalized with the early endosome marker Rab5 (Fig. 1*B*). Consistent with previous studies, NHE9 also localized to early endosomes (Fig. 1*C*). To assess the impact of NHE9 on spike protein trafficking from early to late endosomes, we modified the HEK293T-hACE2 cell line to overexpress the NHE9 protein (referred to as NHE9+). Western blot analysis confirmed a 2.5-fold increase in NHE9 expression in NHE9+ cells compared to control cells (Fig. 1*D*). Given that NHEs transport approximately 1500 ions per second, even a small increase in their expression is physiologically significant (22). Next, we investigated whether the increase in NHE9 affected the delivery of the spike protein to late endosomes by assessing its colocalization with the late endosomal marker Rab7. In NHE9+ cells, spike protein levels in late endosomes were 21% lower than in control cells (Fig. 1, *E* and *F*). To confirm that this result was due to NHE9 overexpression, we performed a knockdown of NHE9 in NHE9+ cells (NHE9+ KD, Fig. 1*D*). This knockdown restored spike protein levels in late endosomes to those comparable to control cells (Fig. 1*F*). This result indicates that the impaired spike protein delivery was indeed due to increased NHE9 expression. Although the levels of NHE9 in the NHE9+ KD cells were slightly lower than in control cells, we did not observe a significant difference in spike protein delivery to late endosomes between the two groups. This could be due to the activation of compensatory mechanisms, as noted in previous studies (23, 24), or it could be outside the detection range of the assay. Nevertheless, these data collectively demonstrate that an increase in NHE9 expression within early endosomes hinders the trafficking of viral cargo to late endosomes.

### Endosomal pH modulation function of NHE9 underlies viral cargo trafficking defects

**Figure 2:**
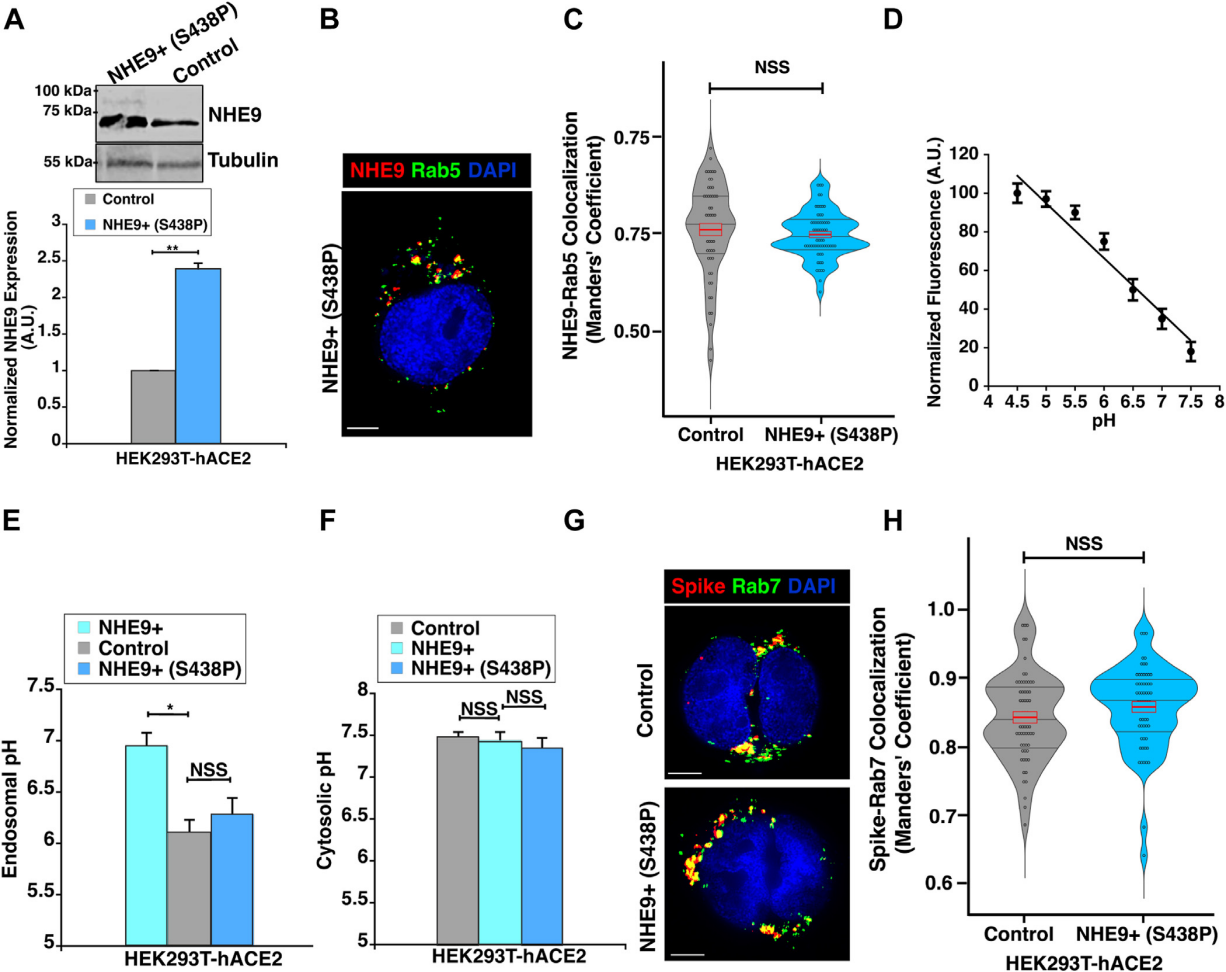
**Regulation of early endosomal pH underlies impaired spike protein delivery to late endosomes.***A*, *Top panel*: Immunoblot showing NHE9 expression in HEK293T-hACE2 cells overexpressing a mutant of NHE9 defective in pH modulation function (NHE9+ (S438P)) compared to control. *Bottom panel*: Graphical representation of the average band intensity from densitometric scans of immunoblots for three biological replicates. Error bars represent standard deviation (SD). ∗∗*p* < 0.005. Statistical analysis was performed using Student’s *t* test. *B*, representative immunofluorescence microscopy image showing the colocalization of NHE9 (*red*) with the early endosomal marker Rab5 (*green*) in NHE9+ (S438P) cells. Colocalization is indicated by *yellow dots*. DAPI staining of the nucleus is shown in *blue*. The scale bar (*white line*) represents 5 μm. *C*, quantitative comparison of the colocalization of NHE9 with Rab5 between control and NHE9+ (S438P) cells. Violin plots show the distribution of Manders' overlap coefficient values, with *red rectangles* indicating the mean (*center line*) and standard deviation (*top and bottom of the rectangles*). The data represents the mean of two biological replicates, each consisting of at least 30 cells. Error bars represent standard deviation (SD). NSS, Not statistically significant. Statistical analysis was done using Student’s *t* test. *D*, calibration of endosomal pH using the fluorescence ratio of internalized Dextran conjugated to a pH-sensitive fluorophore (pHrodo Green) and a pH-insensitive fluorophore (Cascade Blue). Cells were loaded with tagged Dextran and exposed to nigericin (100 μM) in a pH-defined medium ranging from pH 4.5 to pH 8.0. *E*, The pH of early endosomes was determined using a ratiometric method with pH-sensitive Dextran, normalized for differences in uptake using pH-insensitive Dextran. Dextran uptake was conducted for 5 to 7 minutes to ensure pH measurement in early endosomes. Calibration was performed using buffers of known pH as described in (*D*). *F*, cytoplasmic pH was determined by measuring the fluorescence emission of BCECF at pH-sensitive and pH-insensitive wavelengths, calibrated in buffers of known pH. Graphs represent the mean from three biological replicates, each with at least 30 cells. Error bars indicate standard deviation (SD). ∗*p* < 0.05 and NSS, Not statistically significant. Statistical analysis was done using Student’s *t* test. *G*, representative immunofluorescence microscopy images comparing the colocalization of the spike protein (*red*) with the late endosomal marker Rab7 (*green*) between control (*top panel*) and NHE9+ (S438P) (*bottom panel*) HEK293T-hACE2 cells, captured 20 minutes post spike protein internalization. Colocalization is indicated by *yellow dots*. DAPI staining of the nucleus is shown in *blue*. The scale bar (*white line*) represents 5 μm. *H*, quantitative comparison of colocalization of the spike protein with Rab7 between control and NHE9+ (S438P) cells, captured 20 minutes after internalization of the spike protein. Violin plots show the distribution of Manders' overlap coefficient values, with *red rectangles* indicating the mean (*center line*) and standard deviation (*top and bottom of the rectangles*). The data represent the mean of two biological replicates, each consisting of at least 30 cells. Error bars represent standard deviation (SD). NSS, Not statistically significant. Statistical analysis was done using Student’s *t* test.

NHE9 is known for its role in regulating endosomal pH (pH_e_). To determine if the trafficking defects we observed stemmed from NHE9's role in limiting acidification, we utilized the S438P mutant of NHE9, which is defective in pH modulation function (23). We engineered the HEK293T-hACE2 cell line to overexpress the NHE9 mutant protein (referred to hereafter as NHE9+ (S438P)). Western blot analysis confirmed that NHE9 expression increased by approximately ∼2.6-fold in NHE9+ (S438P) cells compared to control cells (Fig. 2*A*). In HEK293T-hACE2 cells, this mutant localized to early endosomes (Fig. 2, *B* and *C*) similar to the wild-type protein. To evaluate the pHe, we used ratiometric imaging with pH-sensitive (pHrodo Green-conjugated) and pH-insensitive (Cascade blue-conjugated) dextrans that are quickly taken up by endocytosis into early endosomes (25). pHrodo green fluorescence diminishes predictably between pH 5 and 8, while Cascade blue remains stable, thus allowing for an accurate normalization of pHe values (26). We calibrated pHe using known pH buffers (Fig. 2*D*). After 5 to 7 minutes of dextran internalization, NHE9+ cells exhibited a 0.84 unit increase in pHe compared to controls (Fig. 2*E*). Despite similar levels of overexpression of wild-type and mutant NHE9 protein (Figs 2*A* and 1*D*), the pHe in NHE9+ (S438P) cells did not show a significant change compared to that in control cells (Fig. 2*E*). There was no significant variance in cytosolic pH among the cells (Fig. 2*F*). Next, to determine if the shift in pHe affects viral cargo trafficking, we monitored the spike protein localization to the late endosomes in NHE9+ (S438P) cells. We assessed its colocalization with Rab7 20 minutes post-internalization. The NHE9+ (S438P) cells exhibited no significant difference in spike protein levels in the late endosomes compared to control cells (Fig. 2, *G* and *H*). These data indicate that NHE9 modulation of pHe is crucial for viral cargo trafficking.

### NHE9 expression modulates PI3P levels and disrupts efficient endosomal transport

**Figure 3:**
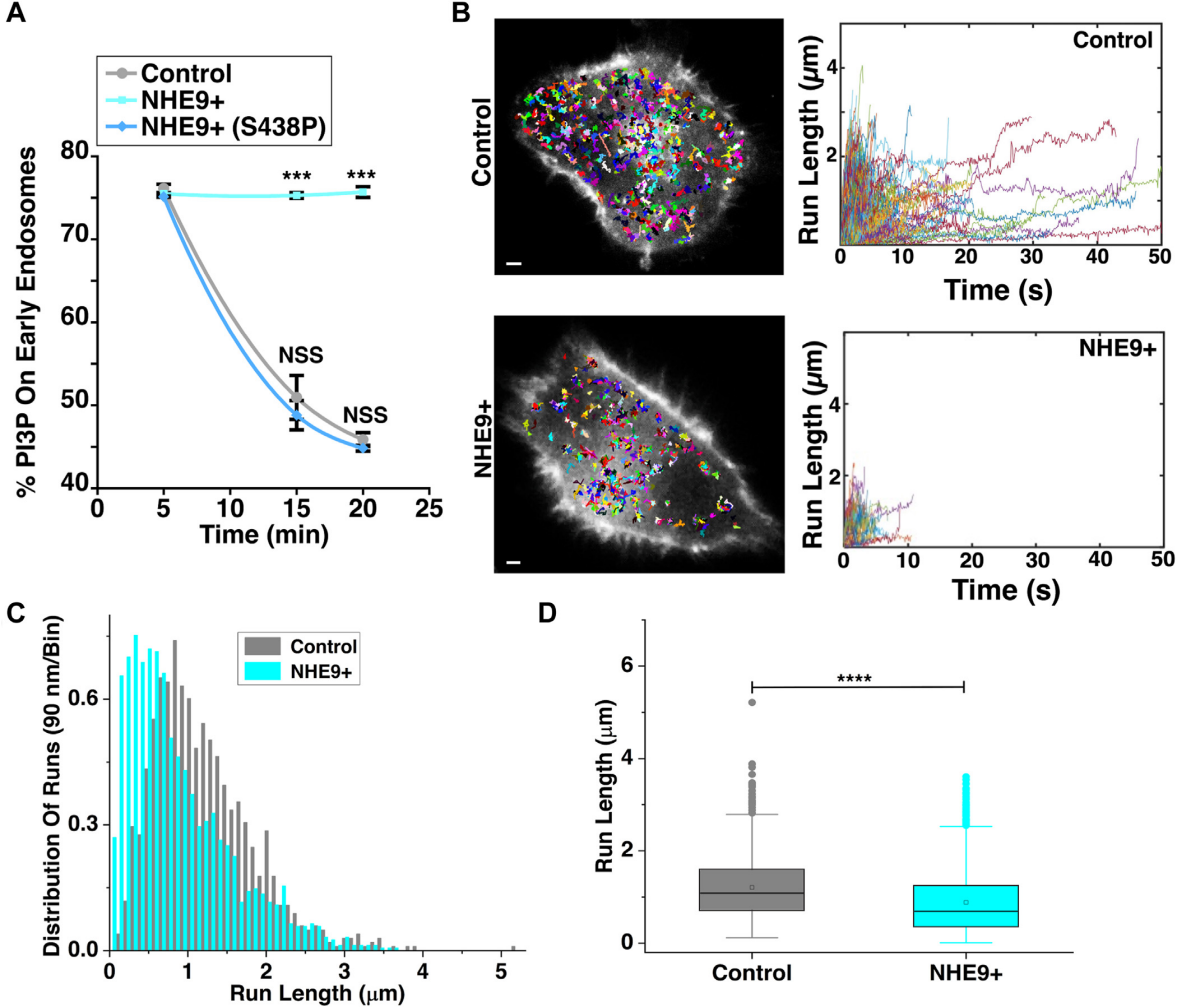
**Changes in luminal pH by NHE9 affect PI3P levels and early endosome movement.***A*, graph showing the levels of PI3P remaining on early endosomes following spike protein internalization for 5, 15, and 20 minutes in control, NHE9+, and NHE9+ (S438P) cells. Quantification was based on Manders' overlap coefficient between PI3P and the early endosome marker Rab5. The plot represents the mean of two biological replicates, each with at least 30 cells. ∗∗∗*p* < 0.001. NSS, Not statistically significant. Statistical analysis was performed using Student’s *t* test. *B*, *Left panel*: representative highly inclined and laminated optical sheet (HILO) microscopy images showing control and NHE9+ cells with endocytosed spike protein tracks. The images were captured at 10 frames per second with a spatial resolution of 83 nm/pixel. Single particle tracking was performed using the 'TrackMate' plugin in Fiji to trace the movements of individual endosomes. *Right panel*: Representative plots showing the radial positions of individual endosomes in control and NHE9+ cells, with movement durations of ≥1 second and a position-time correlation of ≥0.6. Tracks demonstrating net-directional movement toward the perinuclear region were identified based on the condition “End position - Start position <0.” Scale bar (*white line*) is 2 μm. *C*, the figure illustrates the comparative distributions of run lengths of individual endocytosed spike proteins, normalized by the total count, highlighting shifts towards shorter run lengths in NHE9+ cells compared to control cells. *D*, the box plot compares the average run length between control and NHE9+ cells. The plot displays the median, interquartile range, and potential outliers for each sample group. ∗∗∗∗*p* < 0.0001. Statistical analysis was done using ANOVA. Graph represents the mean from three biological replicates and at least 1000 endosomes were used for each. See related Supplemental Fig. S1 and Supplemental Videos 1 and 2.

**Figure 4:**
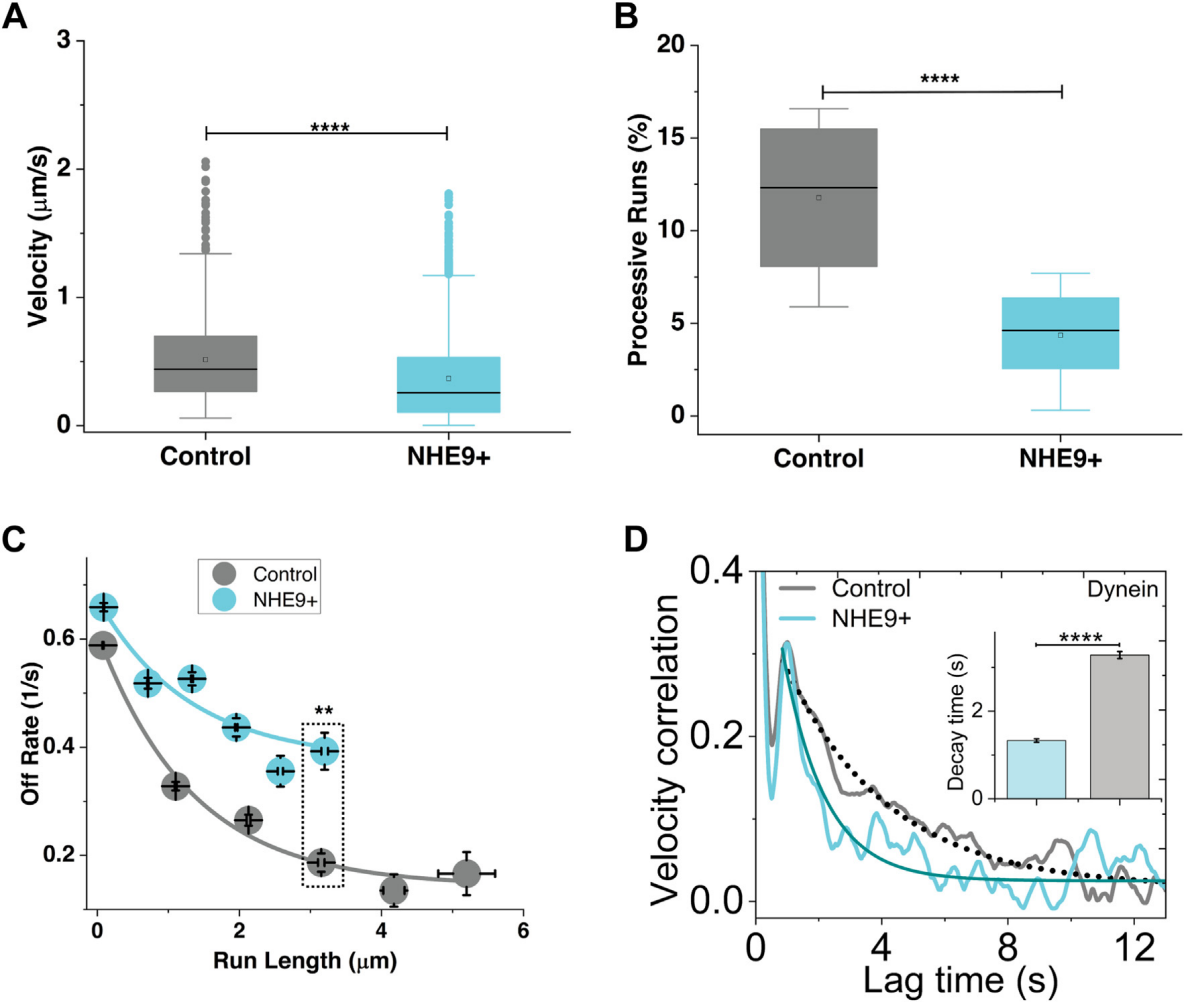
**NHE9 affects the kinetic parameters of early endosomal runs.***A*, the box plots compares the average velocity between control and NHE9+ cells. The plot displays the median, interquartile range, and potential outliers for each sample group. ∗∗∗∗*p* < 0.0001. Statistical analysis was done using ANOVA. *B,* same as *A*, except for percent processive runs. *C*, the graph compares the off-rate (velocity/run length), calculated from the analysis of endosome movement, demonstrating the detachment rate of endosomes from microtubules. Data displayed is the mean ± SEM. ∗∗*p* < 0.01 Statistical analysis was done using ANOVA. Graphs represent the mean from three biological replicates and at least 1000 endosomes were used for each. *D*, the graphs displays the normalized velocity auto-correlation function (VACF), showing the velocity correlation at varying time lags. This figure illustrates how the velocity of cargo at one point in time is correlated with its velocity at another point in time. Statistical analysis for the decay times was done using Student’s *t* test. ∗∗∗∗*p* < 0.0001. Statistical significance for VACF distributions were done using the Nonparametric Kolmogorov-Smirnov Test (*p* < 0.0001).

How do pH changes in the lumen affect early endosome transport by motor proteins in the cytosol? Endosome membrane lipids act as intermediaries, interacting with motor proteins on the cytosolic side and the fluid environment in the lumen (27, 28). Phosphatidylinositol-3-phosphate (PI3P), a lipid specific to early endosome membranes, is essential for regulating motor protein recruitment (29, 30). Previous studies in macrophages have shown that luminal acidification during endophagosome maturation is associated with depletion of PI3P from the vesicle membrane (31, 32). Based on these insights, we hypothesized that luminal pH regulates PI3P levels on early endosomes, thereby impacting motor protein driven transport. To test this hypothesis, we monitored PI3P depletion from early endosomes following spike protein internalization. Control cells showed a typical decrease in PI3P between 5 to 20 minutes (Figs. 3*A* and S1). However, in NHE9+ cells, PI3P persisted beyond 20 minutes (Figs. 3*A* and S1). Cells with the NHE9 (S438P) mutation showed no significant difference in PI3P levels compared to controls (Figs. 3*A* and S1). These findings demonstrate that NHE9-regulated luminal pH modulates PI3P levels on early endosomes. To investigate the impact of this modulation on the kinetic parameters of endosome movement, we utilized highly inclined and laminated optical sheet (HILO) microscopy (33). This technique enabled us to visualize single molecules to a depth of more than several micrometers with enhanced clarity, allowing us to track the trajectory of Alexa Fluor 647-tagged SARS-CoV-2 spike proteins transported by early endosomes (33, 34). We observed that a substantial number of endosomes exhibited directed motion in both control and NHE9+ cells, consistent with microtubule-based transport (Fig. 3*B*, and Supplemental Videos 1 and 2). The distribution of runs exhibited an exponential decrease with increasing run lengths (Fig. 3*C*). NHE9+ cells showed a tendency toward shorter run lengths compared to control cells, indicating that endosomes in NHE9+ cells terminate their movement prematurely (Fig. 3, *B* and *C*). Specifically, the average run length in NHE9+ cells decreased by 27%, from 1.207 μm to 0.885 μm, indicating impaired perinuclear movement of endosomes (Fig. 3*D*). The recorded longest-track lengths were similar between NHE9+ and control cells, indicating that the observed differences in endosomal run lengths were not due to signal decline or recording discrepancies. The average velocity of spike protein transport by early endosomes in NHE9+ cells decreased by ∼30% compared to control cells (Fig. 4*A*). This reduction could be attributed to fewer processive motors on early endosomes in NHE9+ cells. Supporting this, early endosomes in NHE9+ cells exhibited a 67% decline in number of processive runs (Fig. 4*B*) and a 50% higher off rate, implying more frequent detachment from microtubules (Fig. 4*C*). To further understand the early endosome movement, we analyzed the velocity auto-correlation function (VACF) (35). The VACF evaluates how the velocity of an endosome at one time point is correlated with its velocity at a later time. This analysis allows us to gain insights into the persistence of the endosome's motion and the nature of its trajectory. At zero lag (τ = 0), the VACF is normalized to one, indicating perfect self-correlation (Fig. 4*D*). A rapid drop to a lower value within ∼0.5 seconds suggests anti-correlated velocities, indicating the endosome often reverses direction of its trajectory along the microtubule shortly after the initial time. The peak around 1 second indicates a characteristic time scale over which the motors maintain their velocity before changing speed or direction (Fig. 4*D*). Over longer intervals, the correlation drops to zero, showing the motion becomes uncorrelated. Significant differences in early endosome movement towards the cell center were observed between NHE9+ and control cells (Fig. 4*D*-inset). The exponential decay time for NHE9+ cells is 1.33 ± 0.04 seconds, about 2.5 times faster than the 3.28 ± 0.08 seconds in control cells, indicating more random movement in NHE9+ cells compared to consistently unidirectional movement in control cells. Overall, our results indicate that NHE9 expression regulates PI3P levels, thereby impacting the transport kinetics of spike protein by early endosomes.

### NHE9 is a promising target to impair endocytic entry of SARS-CoV-2

**Figure 5:**
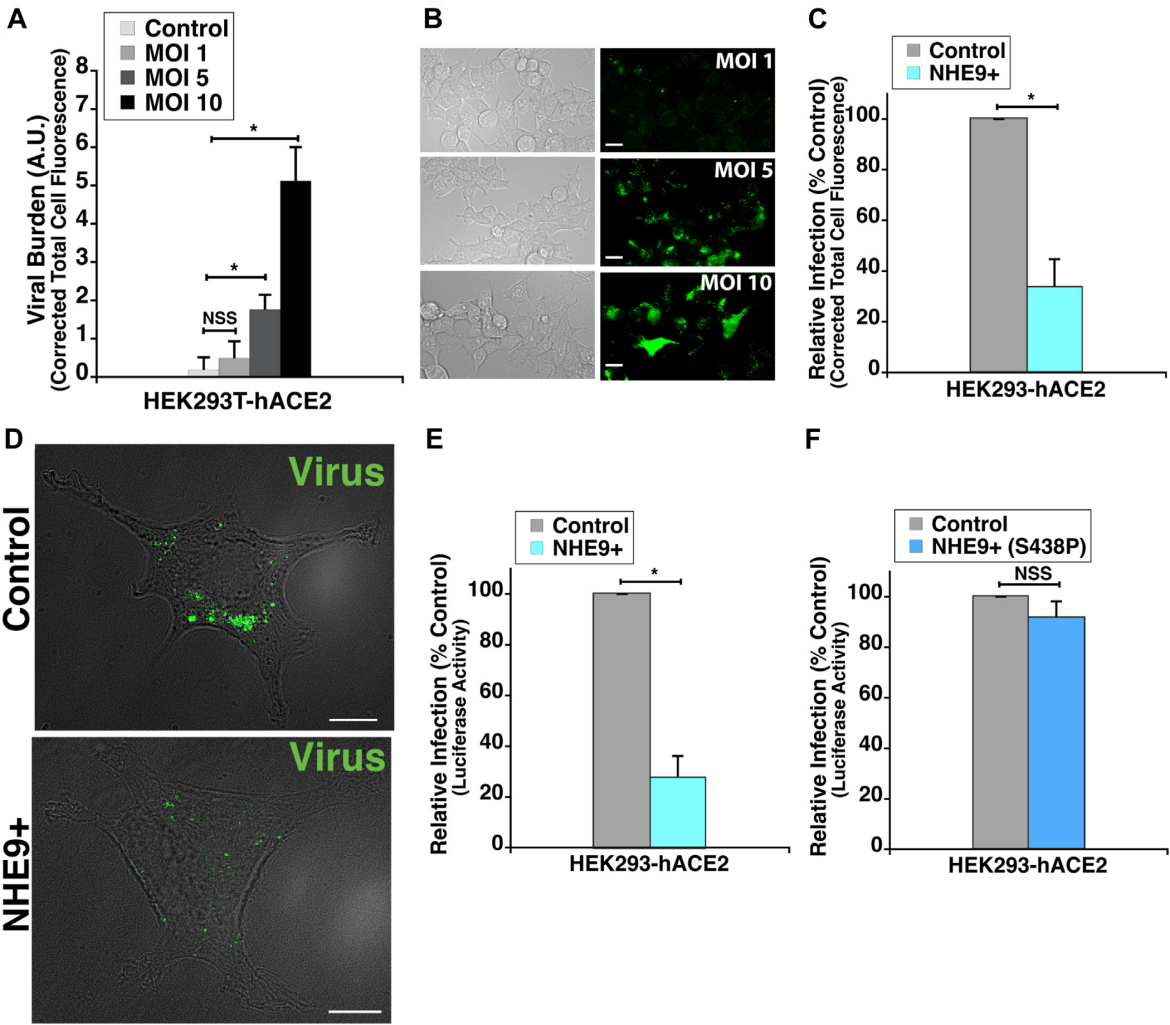
**NHE9 expression modulates entry of SARS-CoV-2 spike-bearing virus in HEK293T-hACE2 cells.***A*, HEK293T-hACE2 cells were infected with SARS-CoV-2 spike protein-pseudotyped lentivirus at various multiplicities of infection (MOI) as indicated. The graph compares Corrected Total Cell Fluorescence (CTCF) data for GFP reporter expression at the specified MOIs. CTCF corrects background fluorescence by subtracting the estimated background contribution (product of the cell area and background mean fluorescence) from the total integrated density of the region of interest. The data represent the mean from three biological replicates, each with at least 30 cells. Error bars represent standard deviation (SD). ∗*p* < 0.05; NSS, Not statistically significant. Statistical analysis was performed using Student’s *t* test. *B*, live cell images showing bright field (left panels) and GFP fluorescence (right panels) at 12 hours post-infection. GFP expression serves as a reporter for viral entry. The scale bar (*white line*) represents 30 μm. *C*, CTCF analysis comparing GFP expression in control and NHE9+ HEK293T-hACE2 cells 12 hours post-infection with SARS-CoV-2 spike-bearing virus. The graph represents the mean from three biological replicates, each with at least 30 cells. *D*, representative bright field images of live HEK293T-hACE2 cells showing GFP expression as a marker for entry of SARS-CoV-2 spike-bearing virus, 12 hours post-infection. The *upper panel* displays control cells, while the *bottom panel* displays NHE9+ cells. The scale bar (*white line*) represents 10 μm. *E*, comparison of the SARS-CoV-2 spike-bearing virus burden in control and NHE9+ HEK293T-hACE2 cells, assessed by luciferase assay. *F*, same as (E) but comparing NHE9+ (S438P) to control cells. Both graphs represent the mean from three biological replicates, each with at least 30 cells. Error bars represent standard deviation (SD). ∗*p* < 0.05; NSS, not statistically significant. Statistical analysis was performed using Student’s *t* test.

**Figure 6:**
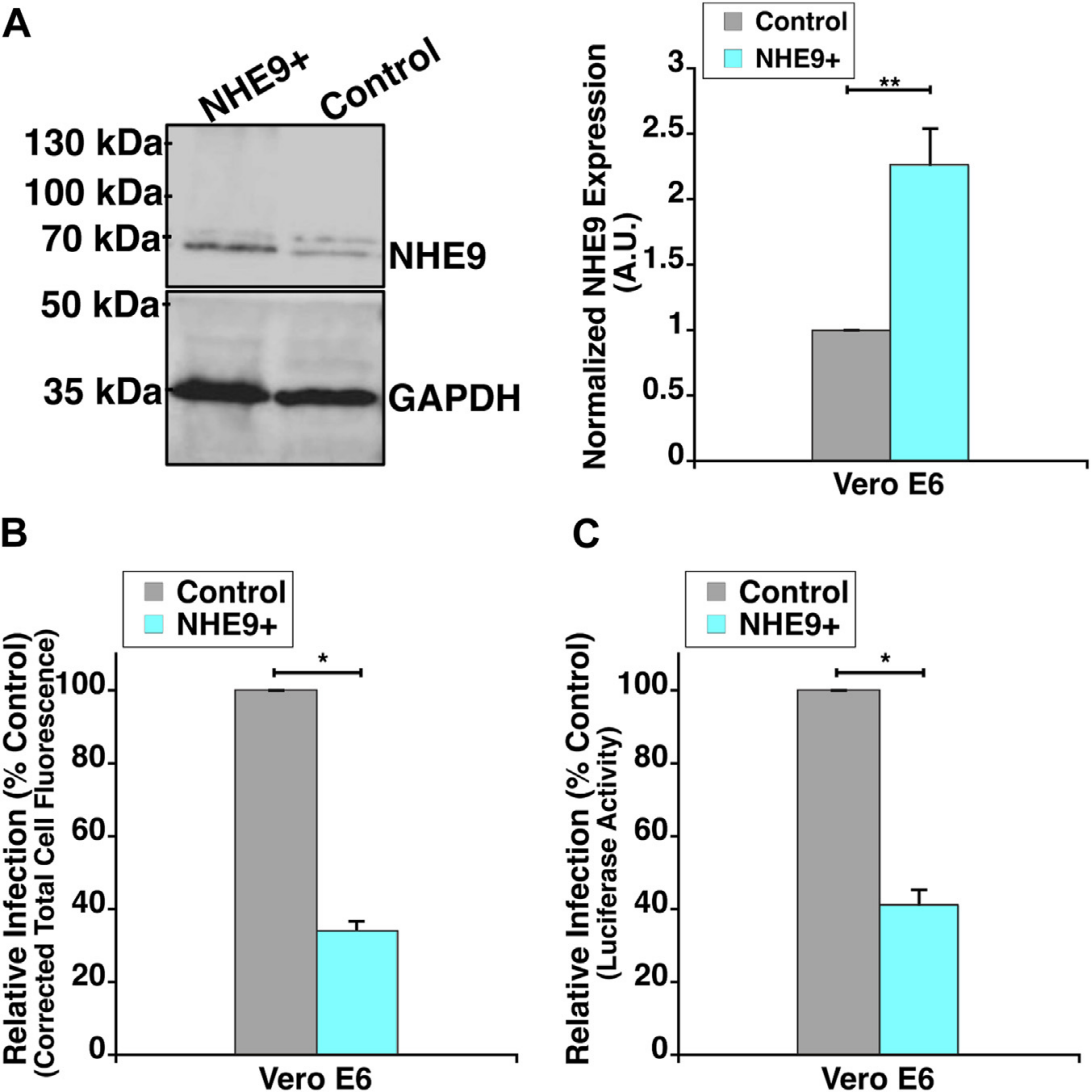
**NHE9 overexpression lowers viral burden of SARS-CoV-2 spike-bearing virus in Vero E6 cells.***A*, *Left panel*: immunoblot showing NHE9 expression in NHE9+ compared to control Vero E6 cells. *Right panel*: Average band intensity, obtained from densitometric scans of immunoblots, graphically represented for three biological replicates. Error bars represent standard deviation (SD). ∗∗*p* < 0.005. Statistical analysis was performed using Student’s *t* test. *B*, corrected total cell fluorescence (CTCF) analysis comparing GFP expression in control and NHE9+ Vero E6 cells 12 hours post-infection with SARS-CoV-2 spike-bearing virus. *C*, comparison of the SARS-CoV-2 spike-bearing viral burden in control and NHE9+ Vero E6 cells, assessed by luciferase assay. Both graphs represent the mean of three biological replicates. Error bars represent standard deviation (SD). ∗*p* < 0.05. Statistical analysis was performed using Student’s *t* test.

Given our findings that NHE9 overexpression reduces the acidification of early endosomes and impairs spike protein trafficking, we investigated its effect on viral infectivity. To this end, we utilized an established method involving a pseudotyped virus that bears the SARS-CoV-2 spike protein and reporter gene sequences (9, 12). HEK293T-hACE2 cells were incubated with these pseudotyped viral particles, which contained GFP and luciferase reporters, at various multiplicities of infection (MOI) for 12 hours. We quantified infectivity by measuring GFP expression and determined that an MOI of 10 was optimal (Fig. 5, *A* and *B*). Therefore, this MOI was used for subsequent studies. To evaluate viral entry, we compared reporter gene expression in control and NHE9+ HEK293T-hACE2 cells using fluorescence microscopy (FM) and luciferase assays (LF). We observed a significant reduction in viral burden within NHE9+ cells, with reductions of approximately 67% (FM) and 72% (LF) compared to controls (Fig. 5, *C*–*E*). Consistent with the trend from spike protein trafficking assays, we observed no significant difference in viral infectivity in NHE9+ (S438P) cells compared to the control (Fig. 5*F*). We further validated our results in Vero E6 cells, which are commonly used for viral infection studies due to their high native ACE2 expression (5). NHE9 was stably overexpressed in Vero E6 cells, leading to a 2.25-fold increase in protein levels (Fig. 6*A*). Similar to HEK293T-hACE2 cells, Vero E6 cells overexpressing NHE9 (NHE9+) showed a comparable decrease in viral burden (Fig. 6, *B* and *C*). These results underscore NHE9's crucial role in modulating viral infectivity and reaffirm its potential as a target to inhibit the endocytic entry pathway of SARS-CoV-2.

## Discussion

When a virus is detected, the innate immune system activates signaling pathways that lead to the production and release of type I interferons (IFNs), primarily IFN-α and IFN-β (36). These interferons establish a strong antiviral state by promoting the synthesis of various antiviral proteins (36). Notably, IFN-β induces the expression of NHE9 (37). Our study demonstrates that NHE9 expression reduces viral burden in host cells by impairing the entry of a SARS-CoV-2 pseudotyped virus. SARS-CoV-2 inhibits IFN signaling pathways and modulates IFN-stimulated gene expression, potentially keeping NHE9 levels low (20, 38, 39). Therefore, upregulating NHE9 through direct genetic perturbation or indirectly *via* IFN-β could potentially protect against viral infection. Targeting the endocytic entry steps of SARS-CoV-2 infection is an effective strategy for limiting viral spread and reducing severity. However, the absence of druggable candidates along the endocytic pathway has hindered the development of therapies. While acknowledging the limitations of using a pseudotyped virus, our study takes a crucial first step in understanding the connection between NHE9 and viral entry.

SARS-CoV-2 recognizes host receptor angiotensin converting enzyme II (ACE2) and potentially other receptors such as CD147 and Neuropilin (NRP1), depending on the host cell type (40). This interaction mediated by the virus’s spike protein induces a rearrangement of cortical actin, membrane bending, and recruitment of endocytic machinery (10). The receptor bound virus is then transported to early endosomes. These newly formed vesicles are transported away from the cell periphery as they switch from actin-to microtubule-based movement (41). Long-range movements are necessary for the endocytosed virus to get to the late endosomes (10). Two types of motors, kinesin and dynein support movement of the endosomes towards the plus and minus ends of the microtubules, respectively (41). The lipid membrane of the endosome plays a significant role in the recruitment and positioning of the motor proteins (42, 43, 44). Motors of both types are typically found on each endosome, where their conflicting actions cause the endosome to move back and forth with frequent changes in direction. This opposing motor activity needs to be regulated in a way that favors movement either predominantly in the positive or negative direction. Our results indicate luminal pH of the early endosomes fine-tuned by NHE9 expression, plays a crucial role in controlling the direction of endosome movement.

**Figure 7:**
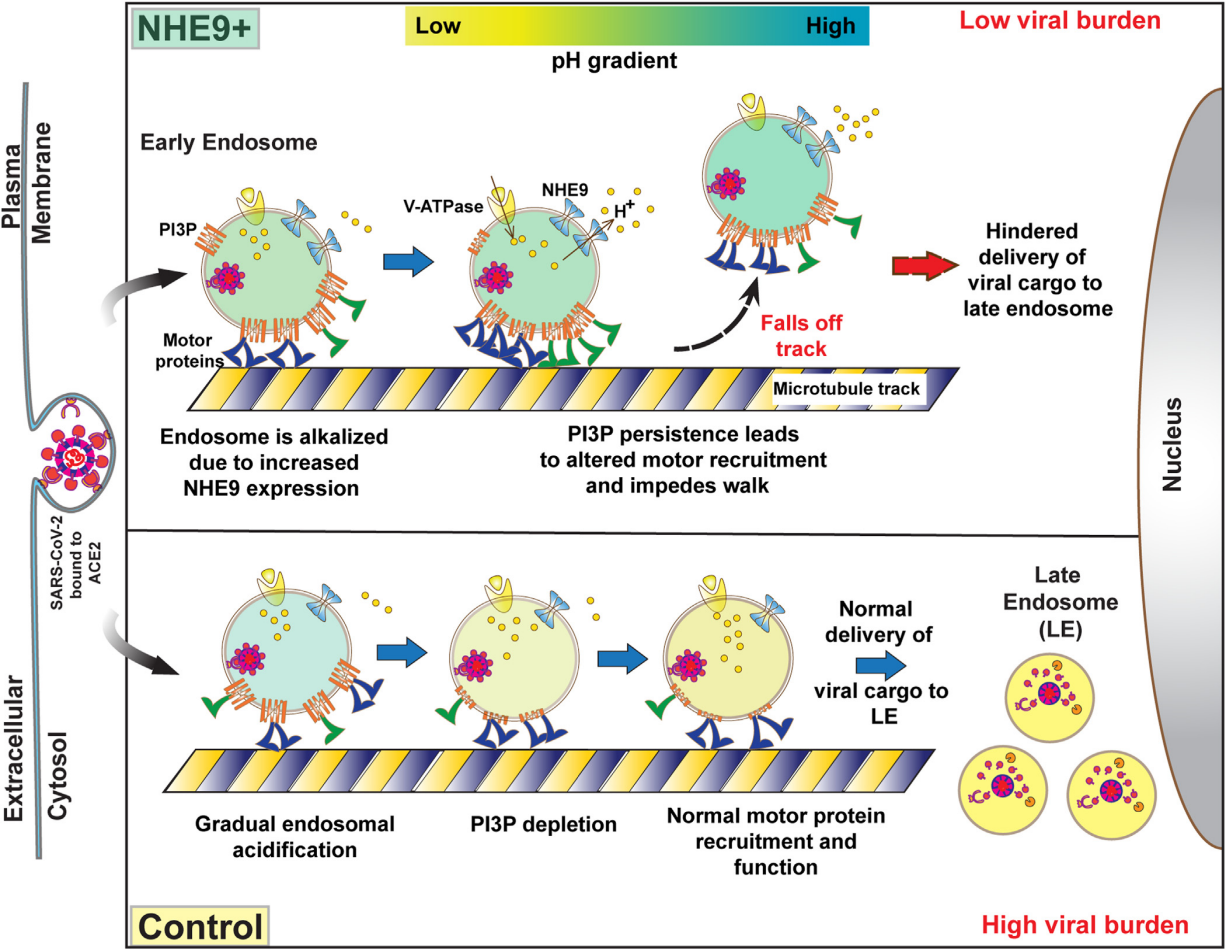
**Schematic illustrating the impairment of SARS-CoV-2 entry into host cells due to NHE9-mediated luminal alkalization.** Early endosomes (EEs) carrying the endocytosed SARS-CoV-2 virus bound to the ACE2 receptor are shown on microtubules. Endosomal acidification is regulated by V-ATPase, which pumps protons into the endosomal lumen, and NHE9 alkalizes the lumen by moving protons out of the endosome. Phosphatidylinositol-3-phosphate (PI3P) on early endosomes plays a crucial role in the recruitment of motor proteins important for the movement of early endosomes towards the late endosomes located at the perinuclear regions of the cell. Under normal conditions (*Control, bottom panel*), as the early endosome matures, its lumen acidifies and PI3P levels on the membrane are depleted over time. This decrease in PI3P levels is essential for the proper maturation and perinuclear trafficking of viral cargo to late endosomes. When the lumen of the early endosome is alkalized due to increased expression of NHE9 (NHE9+, *top panel*), it leads to increased retention of PI3P on early endosome membranes. This plausibly leads to altered recruitment disrupting the balance and coordination of the oppositely directed motor proteins, which rely on tightly regulated cycles of attachment and detachment from microtubules. These cycles are typically influenced by the lipid composition of endosomal membranes and associated regulatory proteins. Disruptions caused by persistent PI3P increase the frequency of detachment from microtubules and decreased run length and velocity of EE movement. This inefficient transport of viral cargo to late endosomes ultimately contributes to a decreased viral burden in host cells.

Motor proteins are recruited to the early endosome membrane through interactions with PI3P, as well as the small GTPases Rab4 and Rab5. Luminal pH controls the accumulation of PI3P (31, 32). The lipid kinase Vps34 generates PI3P on the endosomal membrane. As the compartment matures, luminal acidification causes Vps34 to dissociate from the membrane, halting PI3P synthesis (31, 32). Consistent with this, we show that limiting this acidification *via* increased NHE9 expression prolongs the persistence of PI3P on the endosome membrane. Prolonged PI3P could lead to the excessive recruitment of motor proteins or their adaptors. This overcrowding might create steric hindrance thereby reducing their collective efficacy at engaging with microtubules and sustaining long runs. This is supported by our findings of decreased run lengths and speed for early endosomes moving towards the cell center. Additionally, if both sets of motors are recruited and activated simultaneously, it's plausible that this could lead to an increased tug-of-war situation, where the opposing forces of dynein and kinesin result in erratic movement of the endosomes. Our analysis of the velocity auto-correlation function (VACF) provides evidence of increased competition between opposing motors in NHE9+ cells. We observed significantly quicker velocity decorrelation in NHE9+ cells compared to control cells, indicating that movement becomes more random over time rather than being directed towards the peri-nuclear late endosomes. This suggests increased hindrance to the movement of dynein motors, likely due to counteractive forces from kinesin motor proteins. Finally, persistent PI3P on early endosomes could interfere with the coordination between dynein and microtubule-associated proteins, which are integral in stabilizing and guiding motor protein movement along microtubules. Consistent with this interference, we observed increased off-rates, indicating that the endosomes transporting viral cargo tend to fall off the microtubule tracks in NHE9+ cells. As a result, fewer viral particles are delivered to the late endosomes (Fig. 7), leading to the decreased viral burden we observed. Further experiments investigating the mechanical properties of endosomal motor protein movement are needed to delineate the precise mechanisms involved.

The regulation of endosomal pH involves a complex interplay of multiple proteins (14). Alterations in the levels or activity of one pH-regulating protein can have cascading effects on others, ensuring the maintenance of physiological pH within the endosome (14).This could explain why the trafficking of viral cargo from early endosomes is not completely blocked, at the tested levels of NHE9 expression. Next, NHE9 is not only found on early endosomes but also localizes to late endosomes in most cells (11, 17, 18, 23, 45, 46). Therefore, it cannot be disregarded that NHE9 may affect pH-dependent viral entry processes in the late endosome, such as protease activity and membrane fusion. The reduction in viral burden we observed could indeed be the cumulative outcome of NHE9's impact on pH-dependent steps within both the early and late endosomes.

An important host cell factor regulating SARS-CoV-2 entry is the pH of the endosomes (8). Our findings underscore the importance of early endosome pH in regulating viral entry, highlighting a role that precedes the known pH-dependent steps in viral entry. This positions NHE9 as a potential therapeutic target for modulating endocytic viral entry.

## Experimental procedures

### Cell culture and genetic manipulation

Cultures of HEK293T-hACE2 cells and Vero E6 cells (both from BEI Resources) were maintained in Dulbecco's Modified Eagle's Medium (DMEM; ThermoFisher Scientific) supplemented with 10% fetal bovine serum (Sigma) and 5% antibiotic-antimycotic solution (10,000 units/ml penicillin, 10,000 mg/ml streptomycin; Gibco). Cells were cultured under 5% CO2 at 37 °C. The wild-type NHE9 (NHE9+) and its pH-defective mutant S438P (NHE9+ S438P) were stably overexpressed in HEK293T-hACE2 cells through transfection using Lipofectamine 3000 (Invitrogen), followed by selection with 10 μg/ml blasticidin. NHE9 knockdown (NHE9+ KD) was stably expressed in NHE9+ cells *via* shRNA transfection using Lipofectamine 3000 (Invitrogen) with two specific shRNA clones, pGipZ-SLC9A9-178964 and pGipZ-SLC9A9-178967 (Horizon Discovery), both containing GFP for selection. Controls were transfected with scrambled shRNA controls. NHE9 overexpression in Vero E6 cells was achieved through lentiviral transduction, followed by selection with 10 μg/ml blasticidin. The full-length human SLC9A9 clone was obtained from Genecopoeia.

### Protein extraction and Western blotting

Upon reaching approximately 85% confluency, cells were centrifuged at 1100 rpm for 5 minutes and then resuspended in mammalian protein extraction reagent (Thermo Fisher Scientific, 78,501) supplemented with Halt protease and phosphatase inhibitor cocktails (Thermo Fisher Scientific; 87,785, 78,420). The lysates were then centrifuged at 14,000*g* for 8 minutes at 4 °C. The supernatants were dissolved in Laemmli loading buffer (375 mM Tris-HCl, pH 6.8, 9% SDS, 50% glycerol, 9% β-mercaptoethanol, 0.03% bromophenol blue; Thermo Fisher Scientific). The mixture was heated in a 70 °C water bath for 10 minutes.

Protein samples were separated using SDS-PAGE gel electrophoresis at 100 V for 90 minutes in Novex Tris-Glycine SDS Running Buffer (Thermo Fisher Scientific, LC2675). The gels were then transferred to a PVDF membrane in Tris/Glycine Buffer (25 mM Tris, 192 mM glycine, pH 8.3; Bio-Rad) at 100 V for 90 minutes at 4 °C. The membrane was blocked at room temperature for 60 minutes and then incubated overnight at 4 °C with primary antibodies: 1:100 dilution of SLC9A9 monoclonal antibody (PA5-42524, Thermo Fisher Scientific) and 1:500 dilution of monoclonal α-tubulin antibody (T6199, Sigma) in blocking solution. The membrane was washed with TBST (20 mM Tris, 500 mM sodium chloride, pH 7.5, 1% Tween; Bio-Rad) before and after a 60-minute incubation at room temperature with 1:5000 dilutions of IRDye 680RD and IRDye 800CW secondary antibodies in blocking solution (LI-COR; 926-68071, 925-32210). Primary antibodies were validated using blocking peptides followed by immunofluorescence microscopy, as well as through literature citations where the antibodies were previously utilized. Blots were imaged using the LI-COR Odyssey Fc system and analyzed using ImageJ (47).

### Immunofluorescence microscopy and image analysis

Cells on coverslips were washed with phosphate-buffered saline (PBS) and fixed for 10 minutes at room temperature with 4% paraformaldehyde. After washing with cold PBS, cells were permeabilized with 0.1% Triton-X in PBS containing 1% BSA and 0.3 M glycine for 10 minutes. Cells were then incubated for 1 hour in a blocking solution of 1% BSA and 0.3 M glycine in PBS. For NHE9 staining, cells were incubated overnight at 4 °C with NHE9 antibody (PA5-42524, Thermo Fisher Scientific) at a 1:100 dilution in blocking solution. For ACE2 staining, cells were incubated overnight at 4 °C with the ACE2 antibody (GTX01160, GeneTex) at a 1:100 dilution in blocking solution.

For colocalization studies, cells were treated with fluorescently tagged SARS-CoV-2 spike protein (Recombinant SARS-CoV-2 Spike RBD, AFR10500020, R&D Systems) and incubated on ice for 30 minutes before being transferred to 37 °C. Cells were then fixed at various timepoints as indicated, following endocytosis. Fixed cells were permeabilized, blocked, and incubated overnight at 4 °C with Rab5 antibody (PA5-29022, ThermoFisher Scientific), Rab7 antibody (PA5-52369, ThermoFisher Scientific), or PI3P antibody (Z-P003, Echelon Biosciences) (48, 49, 50). After primary antibody treatment, cells were washed thrice with PBS and incubated with Alexa Fluor-conjugated secondary antibodies (Invitrogen) at 1:500 dilutions for 1 hour. Cells were washed with PBS thrice, treated with DAPI for 5 minutes, and then washed again with PBS before mounting them onto slides using antifade mounting medium (Vectashield, Vector Labs).

Fixed cells were imaged using the epifluorescence setting of the Echo Revolve R4 microscope. The spike protein was imaged in the CY5 channel (excitation: 640/30, emission: 690/50, dichroic mirror (DM): 660), NHE9 and Rab5 in the TxRED channel (excitation: 560/40, emission: 635/60, DM: 600), Rab5/7 and PI3P in the FITC channel (excitation: 470/40, emission: 525/50, DM: 495), and DAPI was imaged in the DAPI channel (excitation: 385/30, emission: 450/50, DM: 425). Images were analyzed by scanning at least 30 cells per test group in at least two independent experiments. Manders’ Overlap Coefficients (MOC) were determined using the Colocalization Finder plugin in ImageJ (51). To quantify the degree of colocalization between the two fluorescent channels, MOC was employed. MOC was chosen for its ability to provide a single, comprehensive metric that reflects the overall overlap between the channels, thus simplifying the comparative analysis across different samples and conditions. Fluorescence intensity was measured by calculating the corrected total cell fluorescence (CTCF) value for each cell, subtracting the product of the cell area and the mean fluorescence of the background reading from the integrated density. Live cell 4imaging was performed using the upright setting of the Echo Revolve R4 microscope. Cells were washed with PBS and treated with live cell imaging media (ThermoFisher) before imaging at room temperature and atmospheric carbon dioxide levels. All image analyses were performed using ImageJ software (47).

### Production of SARS-CoV-2 spike-pseudotyped virus and analysis of viral burden

The pLV-Spike plasmid, encoding the full-length Spike sequence from the original Wuhan isolate, was sourced from Invitrogen. Lentiviral particles carrying the SARS-CoV-2 Spike (S) protein were produced through co-transfection with the pGF1 lentiviral plasmid expressing CMV-GFP-firefly luciferase. When adding viruses to cells in the experiments, the MOI was calculated based on the following formula: MOI=Number of Infectious Viral Particles Added/Number of Target Cells. To achieve a multiplicity of infection (MOI) of 10, ∼1.87 ml of viral stock, with a titer of 4.27 × 10^5^ infectious viral particles per milliliter, was added to 80,000 cells. The viral stock was mixed thoroughly with the cell culture medium to ensure even distribution of virus particles before being added to the cells. The cells were then incubated with the virus under appropriate conditions for the duration of the infection period as specified. For imaging of SARS-CoV-2 spike-pseudotyped lentiviral infectivity, cells were incubated with the virus for 12 hours at 37 °C and moved to live cell imaging solution (Invitrogen) at the time of imaging. Data analysis, including corrected total cell fluorescence (CTCF) and colocalization using MOC, was performed as described previously in the Microscopy and Image Analysis section. Luciferase assays were conducted using a Promega luciferase assay kit, following the manufacturer's instructions as previously described.

### pH measurements

Endosomal acidification was detected with ratiometric imaging using pH-sensitive dextran (pHrodo Green, ThermoFisher, P35368) and pH-insensitive dextran (Cascade Blue, ThermoFisher, D1976), both at concentrations of 75 μg/ml, according to the manufacturer’s instructions. Cytosolic pH was determined by measuring BCECF fluorescence (Life Technologies) as described previously (18).

### Motility of spike protein

#### Sample preparation for highly inclined and laminated optical sheet (HILO) microscopy

HEK293T-hACE2cells were seeded onto poly-L-lysine-coated FluorDishes (World Precision Instruments). After a 3-hour serum-free incubation, the cells were exposed to the spike protein (Recombinant SARS-CoV-2 Spike RBD, R&D Systems). The spike protein (2 μg) was diluted in 200 μl of DMEM, added to the cells, and incubated on ice for 30 minutes. Following this, the cells were incubated at 37 °C for 5 minutes, washed with DMEM, and then imaged in live cell imaging media (Invitrogen).

#### Detection of endosomal movements using HILO microscopy

Single endosomes in cells were visualized using HILO microscopy (33, 34, 52). Fluorescently tagged SARS-CoV-2 spike protein (Alexa Fluor 647) on endosomes was excited using a 640 nm laser with an oil-based 60× 1.49 NA TIRF objective, 2× optical magnification, and an exposure time of 100 milliseconds. The diameter of the illuminated area was kept constant. The angle of incidence of the excitation laser was adjusted to allow distinct and bright areas up to ∼2 to 3 μm above the coverslip, facilitating the detection of endosomal movement with reduced background noise. Time-lapse images were acquired at 10 frames per second for 20 minutes for each condition. To determine the position of the nucleus, differential interference contrast (DIC) images were taken every 30 seconds.

Movement of single endosomes in cells was tracked using the single-particle tracking plugin “TrackMate” available on Fiji and with provided MATLAB scripts (53, 54). Before tracking, bleaching correction and contrast enhancement were performed using Fiji. For TrackMate, the LoG (Laplacian of Gaussian) detector and the Simple LAP (Linear Assignment Problem) Tracker were employed with the following parameters: a maximum linking distance of 4 pixels, a gap-closing distance of 3 pixels, and a frame gap of 2 pixels (where 1 pixel = 83 nm) (54). The tracks were then analyzed using custom-made LabVIEW programs. To identify processive movement, tracks were selected if they had 15 or more points per track and a run-length to time correlation coefficient of 0.6 or higher. Additionally, tracks were categorized based on their directional movement towards the nucleus, using the condition (End position minus Start position) < 0. The statistical significance of the average run-length, speed, and duration of endosome movement between control and NHE9+ cells was determined using ANOVA and Student’s *t* test.

The velocity autocorrelation function (VACF) calculation method involves several key steps: preprocessing of the input data files, calculation of instantaneous velocities, computation of the VACF, and subsequent averaging across multiple tracks. The analysis was conducted specially for the tracks moving towards center of cell. The time-series data from each particle track, which included the frame number, and the x and y positional coordinates. were converted to velocities by differentiating the position with respect to time, yielding velocity components in the x and y directions. The magnitude of the velocity was then obtained as $v\left(t\right)=\sqrt{v_{x}{\left(t\right)}^{2}+v_{y}{\left(t\right)}^{2}}$. The VACF was computed for each track by evaluating the temporal correlations of the velocity vectors using the equation:

$$\text{VACF}\;\left(\tau \right)=\frac{\langle v\left(t\right)\cdot v\left(t+\tau \right)\rangle }{\langle v{\left(t\right)}^{2}\rangle }$$

Where τ is the lag time, v(t) and v(t+τ) are the velocities at times t and t+τ respectively, and ⟨⋅⟩ denotes averaging over time for each track. This normalization by the mean square velocity ensures dimensionless values for VACF. The VACF was computed up to a lag equal to the length of the shortest track, and individual VACF results from all tracks were then averaged to obtain a global VACF. To enhance the precision and reliability of the results, smoothing techniques were applied to the velocities before correlating them to reduce noise. Additionally, robust outlier detection and removal techniques ensured the high quality of the velocity data used in the VACF calculation.

## Data availability

The authors confirm that the data supporting the findings of this study are available within the article and its supporting information.

## Supporting information

This article contains supporting information.

## Conflict of interest

The authors declare that they have no conflicts of interest with the contents of the article.

## Figures and Tables

**Figure:**
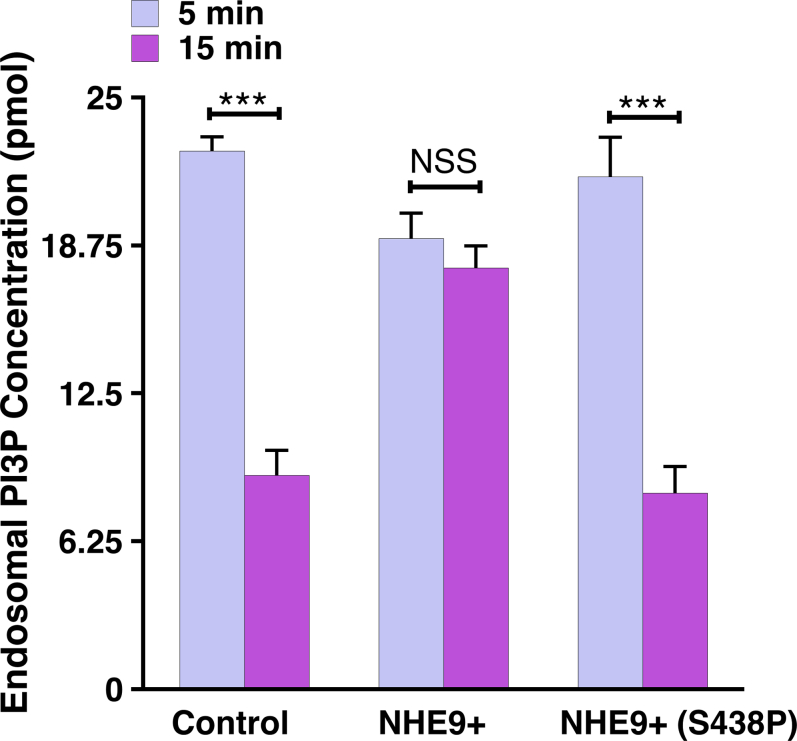
Figure S1
