# Supplementary material for: Acidic pH of early endosomes governs SARS-CoV-2 transport in host cells
Source: J Biol Chem. 2024 Dec 26;301(2):108144. doi: 10.1016/j.jbc.2024.108144 (PMC11815683; doi:10.1016/j.jbc.2024.108144)
Supplement: Supporting Information [file mmc1.docx]

**Supporting Information**

## 1. Legends

- **Fig. S1**: Bar graphs show the average concentrations of phosphatidylinositol-3-phosphate (PI3P) on endosomes in control, NHE9+, and NHE9+ (S438P) HEK293T-hACE2 cells, measured at 5 and 15 minutes after internalization of the endocytic cargo, dextran. Data represent the averages from three biological replicates. Error bars indicate standard deviation (SD). Statistical significance is indicated as follows: ***p < 0.0001; NSS: Not statistically significant. Statistical analysis was performed using Student’s t-test.
- **Videos 1 and 2:** Representative videos obtained through highly inclined and laminated optical sheet (HILO) microscopy show the movement of endocytosed spike proteins in both control cells (vid-1) and NHE9+ cells (vid-2). The videos are presented at 10 times their original recording speed.

##### 2. Methods

**PI3P quantification by ELISA**

Early endosomes were isolated following the manufacturer’s protocol (ED-028, Invent Biotechnologies Inc.) at 5 and 15 minutes following dextran endocytic internalization in equal amounts of control, NHE9-overexpressing (NHE9+), and NHE9+ (S438P) cells. Total protein concentrations were quantified using a BCA protein assay kit (23225, Thermoscientific) to ensure equal amounts of endosomal protein across samples. PI3P was then extracted from the isolated endosomes and quantified using the PI3P Mass ELISA Kit (K-3300, Echelon Biosciences) according to the manufacturer’s instructions. A standard curve was generated by plotting absorbance at 450 nm against known concentrations of PI3P (0.1–1000 pmol), using non-linear regression analysis in GraphPad Prism software, employing a sigmoidal dose response (variable slope) curve with four-parameter logistic (4PL) analysis. Endosomal PI3P concentrations were calculated using the equation derived from this analysis.
